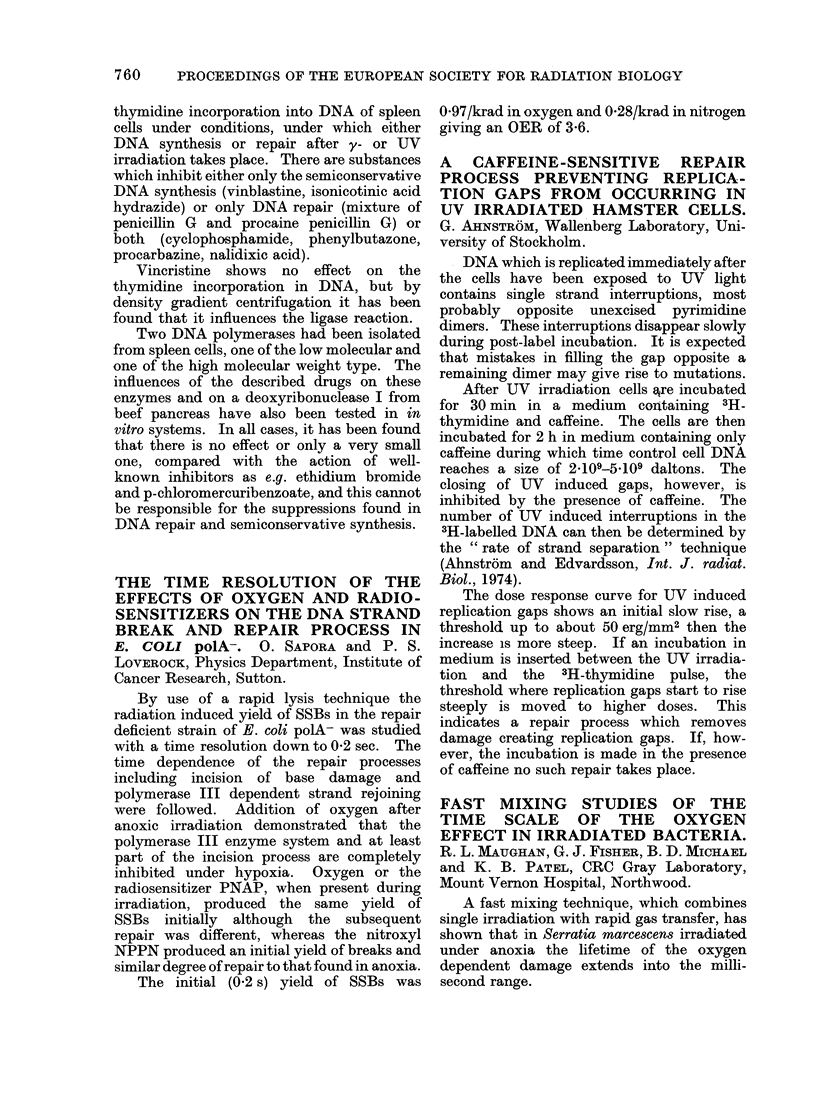# Proceedings: A caffeine-sensitive repair process preventing replication gaps from occurring in UV irradiated hamster cells.

**DOI:** 10.1038/bjc.1975.320

**Published:** 1975-12

**Authors:** G. Ahnström


### A CAFFEINE-SENSITIVE REPAIR PROCESS PREVENTING REPLICATION GAPS FROM OCCURRING IN UV IRRADIATED HAMSTER CELLS.

G. Ahnström, Wallenberg Laboratory, University of Stockholm.

DNA which is replicated immediately after the cells have been exposed to UV light contains single strand interruptions, most probably opposite unexcised pyrimidine dimers. These interruptions disappear slowly during post-label incubation. It is expected that mistakes in filling the gap opposite a remaining dimer may give rise to mutations.

After UV irradiation cells are incubated for 30 min in a medium containing $^3$H-thymidine and caffeine. The cells are then incubated for 2 h in medium containing only caffeine during which time control cell DNA reaches a size of $2{\cdot}10^9$–$5{\cdot}10^9$ daltons. The closing of UV induced gaps, however, is inhibited by the presence of caffeine. The number of UV induced interruptions in the $^3$H-labelled DNA can then be determined by the " rate of strand separation " technique (Ahnström and Edvardsson, *Int. J. radiat. Biol.*, 1974).

The dose response curve for UV induced replication gaps shows an initial slow rise, a threshold up to about 50 erg/mm$^2$ then the increase is more steep. If an incubation in medium is inserted between the UV irradiation and the $^3$H-thymidine pulse, the threshold where replication gaps start to rise steeply is moved to higher doses. This indicates a repair process which removes damage creating replication gaps. If, however, the incubation is made in the presence of caffeine no such repair takes place.